# Supernumerary Teeth

**Published:** 1859-11

**Authors:** 


### SUPERNUMERARY TEETH.

Why some persons have a greater number of teeth than the natural allotment was long considered a mystery ; but it is now cleared up by the *American Dental Review,* which suggests that it is owing to a redundancy of the phosphates, or at least that, in such cases, " there is evidently no lack of the phosphates." We did not suppose that a redundancy would increase the number of the organs ; but, if the doctrine be correct, we should be cautious in the use of these salts, lest, by an increased number of shin bones and their appendages, we become changed from bipeds to quadrupeds.

The editor inquires, in conclusion, if Dr. Watt would neutralize the redundancy in such eases. Certainly he would. If a man is too short, stretch him, and if too long, cut him off, is our motto.

